# Diabetes-Specific Nutrition Formulas in the Management of Patients with Diabetes and Cardiometabolic Risk

**DOI:** 10.3390/nu12123616

**Published:** 2020-11-25

**Authors:** Jeffrey I. Mechanick, Albert Marchetti, Refaat Hegazi, Osama Hamdy

**Affiliations:** 1 The Marie-Josee and Henry R. Kravis Center for Cardiovascular Health at Mount Sinai Heart, Metabolic Support, Division of Endocrinology, Diabetes and Bone Disease, Icahn School of Medicine at Mount Sinai, New York, NY 10029, USA; jeffrey.mechanick@mountsinai.org; 2 Medical Education and Research Alliance (Med-ERA, Inc.), Pompano Beach, FL 33069, USA; 3 Preventive Medicine and Community Health, Rutgers New Jersey Medical School, Newark, NJ 07103, USA; 4 Research and Development Department, Abbott Nutrition, Columbus, OH 43219, USA; refaat.hegazi@abbott.com; 5 Faculty of Medicine, Mansoura University, Mansoura 35516, Egypt; 6 Obesity Clinical Program and Inpatient Diabetes Program, Joslin Diabetes Center, Harvard Medical School, Boston, MA 02215, USA; osama.hamdy@joslin.harvard.edu

**Keywords:** nutrition, therapy, diabetes-specific nutrition formula, diabetes, type 2 diabetes, cardiovascular, cardiometabolic, adiposity, dyslipidemia, dysglycemia

## Abstract

Food-based dietary management, enhanced with evidence-based commercial products, such as diabetes-specific nutrition formulas (DSNFs), can help control the development, progression, and severity of certain chronic diseases. In this review, evidence is detailed on the use of DSNFs in patients with or at risk for diabetes and cardiometabolic-based chronic disease. Many DSNF strategies target glycemic excursions and cardiovascular physiology, taking into account various elements of healthy eating patterns. Nevertheless, significant research, knowledge, and practice gaps remain. These gaps are actionable in terms of formulating and testing relevant and pragmatic research questions, developing an educational program for the uniform distribution of information, and collaboratively writing clinical practice guidelines that incorporate the evidence base for DSNF. In sum, the benefits of DNSF as part of validated clinical practice algorithms include mitigation of chronic disease progression, cost-savings for the healthcare system, and applicability on a global scale

## 1. Introduction

Diabetes-specific nutrition formulas (DSNFs) are specialized forms of therapy that consist of macro- and micronutrient ingredients to manage malnutrition, dysglycemia, and other cardiometabolic risk factors. These formulas have low glycemic indices and complement dietary recommendations for patients with type 2 diabetes (T2D). They contain fiber, monounsaturated fatty acids (MUFAs) and/or polyunsaturated fatty acids (PUFAs), proteins, vitamins, and minerals in palatable, calorie-controlled portions that are used as (1) iso- or hypocaloric meal or snack replacements, (2) hypercaloric supplementation for malnourished patients, (3) very-low-calorie diets, and (4) enteral nutrition support, to an extent determined by clinical circumstances and the discretion of prescribing healthcare professionals.

**Figure 1:**
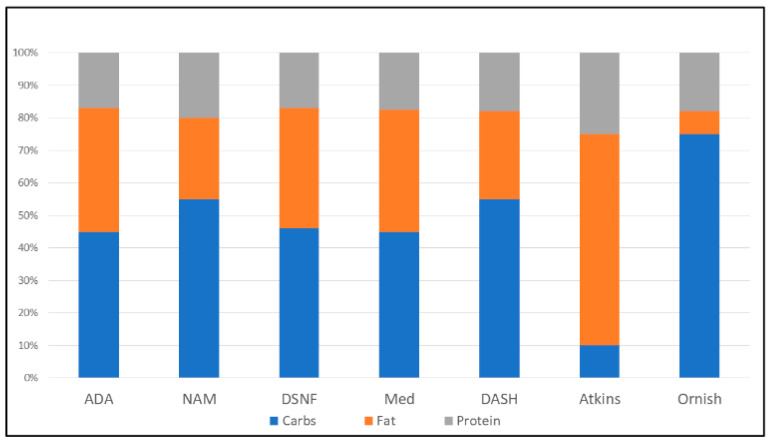
Macronutrient distributions in contemporary eating patterns, diabetes-specific nutrition formulas, and popular diets.

Population-based macronutrient intake data and recommendations for healthy eating can be found in dietary advisories and practice guidelines for general nutritional management as well as the care of patients with T2D and cardiovascular disease (CVD) [1,2,3]. DSNFs generally have macronutrient distribution ranges approximating those in guideline recommendations as a percentage of total calories [4,5,6], while some popular diets bias macronutrient content to prioritize single physiological targets (Figure 1) [1,2,3,4,5,6]. Others address comprehensive cardiometabolic risk mitigation and long-term health. Based on strong epidemiological and mechanistic scientific data, Mechanick et al. [7] configured primary drivers (genetics, environment, and behavior), metabolic drivers (abnormal adiposity, dysglycemia, and other metabolic syndrome traits, with insulin resistance as a critical event), and cardiovascular disease (coronary heart disease, heart failure, and atrial fibrillation) into an actionable framework referred to as cardiometabolic-based chronic disease (CMBCD). Likewise, dysglycemia-based chronic disease (DBCD) conceptualizes a continuum of disease states beginning with insulin resistance and progressing to prediabetes, T2D, and vascular complications; while adiposity-based chronic disease (ABCD) addresses the amount, distribution, and function of body fat—not just the state of obesity. These concepts unify separate components of evolving pathologic processes into diseases with multiple opportunities and pathways for prevention and treatment. In this review, healthy eating principles are discussed to contextualize DSNF evidence and present implications for diabetes care as part of comprehensive CMBCD management. The ultimate objective is to improve therapeutic nutrition for patients with CMBCD while simultaneously prompting (1) further scientific inquiry to close research gaps, (2) educational programs to close knowledge gaps, and (3) the development of infrastructure to close any remaining practice gaps.

The depicted micronutrient proportions (carbohydrates:fats:proteins, respectively) (Figure 1) are computed from reported ranges in diets of patient with diabetes (ADA—45%:20–35%:15–20%) and in the general population (NAM – 45%–65%:20%–30%:10%–30%), in DSNFs (37–55%:30–45%:15–19%, averages from 4 clinically studied common formulas [8,9,10,11]) and in popular diets (Med—40–50%:35–40%:15–20%; DASH—55%:27%:18%; Atkins—10%:60–70%:20–30%; and Ornish—75%:7%:18%).

## 2. Healthy Eating, Diabetes, and Cardiometabolic Risk

Nutrition is the interface between dietetics (the environment) and metabolism (the body), requiring individualization based on nutrigenomic, cultural, environmental, and metabolic parameters. In different populations and cultures, epidemiological, preclinical, and clinical trial data have identified specific foods and eating patterns that are beneficial and form the basis for healthy eating. Macronutrient proportions can be adjusted quantitatively and/or by specific nutrients within each major group for various nutritional purposes.

Dietary manipulation and related assessments are not without complications, however. Modifications in nutrient content can lead to beneficial outcomes based on certain objectives and measures but may also have unintended or even negative consequences that escape detection for lack of consideration or comprehensive analysis. Simply put, individual nutrients cannot be fully assessed in a vacuum; a change in one may effect changes in others and, therefore, confound study results. This conundrum must be considered in all of the discussions that follow.

### 2.1. Carbohydrates

Diets low or very low in carbohydrate content can reduce hemoglobin A1c (A1C) and lower requirements for antihyperglycemic therapies [12]. In a meta-analysis of 11 parallel-group randomized controlled trials (RCTs), low-carbohydrate dieters experienced significantly greater weight loss and high-density lipoprotein cholesterol (HDL-c) levels but also greater low-density lipoprotein cholesterol (LDL-c) concentrations for up to two years [13]. Other meta-analyses of low-carb eating-patterns corroborate these findings [14].

The metabolic effects and clinical results associated with dietary carbohydrates relate not only to total quantities consumed but also their chemical structures. Simple short-chain sugars, such as mono- and di-saccharides with high glycemic indices, are easily digested and quickly absorbed, leading to higher postprandial blood glucose and insidious metabolic problems following continuous long-term consumption. Starch, a complex carbohydrate mixture of amylose composed of several thousand glucose units in straight 1–4 alpha-linked chains and amylopectin consisting of more than 100,000 glucose units in branched 1–6 alpha-linked chains, is absorbed slowly with less glucose excursion. Meals laden with high glycemic-index foods adversely affect T2D and CMBCD risks, while complex carbohydrates with low glycemic indices exert cardiometabolic benefits [15]. Dietary fiber (e.g., cellulose, beta-glucans, and oligosaccharides, as well as other polysaccharides including resistant starches, dextrins, and pectins) refers to a group of straight- and branched-chained carbohydrates (polymers with more than two monomeric units) that are neither digested nor absorbed by the gut as intact molecules. Fiber intake is associated with anti-inflammatory, antioxidant, hypocholesterolemic, hypoglycemic, and antihypertensive effects, verified by RCTs and meta-analyses [16]. Quantitatively, for each incremental gram of daily fiber, a 14% reduction in coronary-event risk (0.86 relative risk; 95% CI, 0.78–0.96) and a 27% decrease in coronary-death risk (0.73 relative risk; 95% CI, 0.61–0.87) have been observed [17].

### 2.2. Fats

As with carbohydrates, dietary fats play contrary roles in CMBCD development and mitigation, depending on both the quantity and kind of SFA consumed. Retrospective data from 11 cohort studies were used to evaluate participants (*N* = 344,696) who substituted MUFA, PUFA, or carbohydrate for dietary SFAs and were followed for 4 to 10 years [18]. A 5% energy substitution of PUFA for SFA, reduced coronary events (HR: 0.87; 95% CI: 0.77, 0.97) and deaths (HR: 0.74; 95% CI: 0.61, 0.89). Similarly, another analysis of pooled data from eight RCTs found that CHD risk declined by 10% for each 5% energy exchange of PUFA for SFA that was maintained for at least one year [19]. In another large study, fat reduction and/or modification of fat type reduced CVD events by 14% (RR 0.86, 95% CI 0.77 to 0.96 for 24 comparisons in 65,614 participants) [20]. Additionally, a double-blind, cross-over RCT assessing five diets of varying SFA proportions showed that a high-MUFA diet was preferable to a low-fat diet due to greater reduction in CVD risk [21].

### 2.3. Proteins

The third major macronutrient component in all human diets is protein. Typically, adults require 1.0–1.5 g of protein per kg body weight per day or about 15–20% of total caloric intake [3]. Following consumption, protein is reduced to its amino acid constituents, absorbed, then reassembled into various forms to meet a variety of physiological needs. Independent of dietary intake, factors such as host metabolism and genetic or enzyme variability may contribute to differences in amino acid concentrations throughout the body. Dysregulated metabolic signaling may also alter amino acid metabolism and concentrations in blood and tissues.

Metabolite profiling has linked certain amino acids to cardiometabolic risks [22]. In patients with obesity, hyperaminoacidemia has been associated with increased insulin secretion in the face of resistance. Specifically, tyrosine and phenylalanine were elevated and correlated with higher insulin concentrations and FFA levels. Levels of lysine, tryptophan, valine, and other amino acids have also been variably correlated with markers of insulin resistance, insulin secretion, and/or risk of diabetes and CVD [23,24].

### 2.4. Phytonutrients

Other than carbohydrates, fats, and proteins, plant-based foods deliver a vast array of additional nutrients, including phytonutrients, such as phenols and terpenes, which may also influence cardiometabolic risk. Polyphenols may decrease absorption and digestion of consumed carbohydrates via inhibition of α-amylase and -glycosidase, as well as vascular cell adhesion molecule, which also participates in early inflammatory events of atherosclerosis [25]. Additionally, plasma insulin levels, hepatic glycogen synthesis, and glucokinase activity have been shown to increase significantly (*p* < 0.05), while blood glucose levels decreased significantly (*p* < 0.05) in response to polyphenol consumption in rodent models [26]. Polyphenols decreased blood glucose in animals with hyperglycemia, protected β-cells against oxidative stress, limited apoptosis, and improved insulin action via changes in adiposity, gene expression, and enzymatic activity [27]. They also inhibited the expression and action of endothelial nitric oxide synthase, thereby facilitating vasorelaxation and decreased LDL-c oxidation, thus offering cardio- and vasoprotection [28]. Moreover, some functional foods containing sterols, stanols, monacolin K from red yeast rice, berberine, beta-glucans, and others nutrients can effectively lower plasma LDL cholesterol levels by about 5–25%, and extend benefits in terms of fatal/nonfatal coronary events, stroke, and all-cause mortality (−31%, −44% and −32%, respectively) [29]. Together, these findings suggest that phytonutrients may be useful in preventing and treating CMBCD and diabetes, which deserves further investigation and consideration for clinical applications.

### 2.5. Micronutrients

Vitamins, minerals, trace elements, and organic acids constitute micronutrients in the human diet that initiate hormone production and accelerate metabolic processes (Table 1) [30,31,32,33,34,35,36,37]. They influence membrane potentials, mitochondrial activities, enzymatic actions, immune mechanisms, neuro-conduction, and muscle contraction to name just a few of their ubiquitous functions [30,31,32,33,34,35,36,37,38]. While acting as cofactors or components of enzyme systems, they potentiate the actions of insulin through activation of receptor sites, enhancement of insulin sensitivity, and prevention of tissue peroxidation. They also support the retention of lean body mass.

Micronutrient deficiency states (Table 2) [38,39,40,41,42] are common and, paradoxically, noted especially among the overweight and obese [38,39,40,41,42]. This evolving trend is sustained by genetic dilution (e.g., genetic manipulation to favor growth vs. nutrient value of agricultural products), environmental dilution (e.g., soil nutrient depletion from excessive use or failure to rotate crops), changes in farming methods (e.g., extensive use of chemicals and fertilizers), and excessive food processing/unhealthy preparation; all leading to premade, overly refined, highly calorie, low-cost, fatty/sugary foods that are greatly reduced in nutritional value [43]. Add to these contributors poor food choices made by consumers across cultures as well as poor dietary planning and eating habits [44] to create a growing population of obese individuals with micronutrient deficiencies as cited in Table 1 and Table 2.

These nutritional deficits have been associated with a multitude of metabolic disturbances that include increased oxidative stress, inflammation, and immune abnormalities [30,31,32,33,34,35,36,37,38,39,40,41,42]. In dysglycemia-based chronic disease, nutritional deficiencies are linked to the progression of β-cell dysfunction and apoptosis, to loss of islet cell mass, and then to the impairment of insulin signaling with compensatory hyperinsulinemia [45]. In adiposity-based chronic disease, nutritional deficiencies play a role opposing lean body mass and maintaining the progression of overweight/obesity to insulin resistance, hypertension, and dyslipidemia, followed by T2D and CVD [30,31,32,33,34,35,36,37,38,39,40,41,42,46]. Although evidence in this field is evolving rapidly, inconclusive and even conflicting research results cloud a precise understanding of mechanisms, relationships, and outcomes. The nature of associations among various micronutrient deficiencies and CMBCD remains unclear.

## 3. Evidence Base for DSNF, Diabetes, and Cardiometabolic Risk

### 3.1. Impact of DSNF on Glycemic Status

The American Diabetes Association and other professional organizations include DSNF in their clinical practice guidelines for patients with diabetes [3]. This is based on the weight of evidence, particularly influenced by RCTs and meta-analyses, demonstrating the benefits of DSNF in various diabetes and cardiometabolic scenarios (Table 3) [4,47,48,49,50,51,52,53]. Through years of research, the use of DSNF has consistently been shown to improve postprandial glucose levels compared to standard test foods such as oatmeal of similar caloric content, either directly through β-cell stimulation and insulin release and/or indirectly through glucagon-like peptide-1 (GLP-1) secretion [48]. In a weight-reduction study, two assayed satiety hormones, peptide YY and glucagon, increased more responsively to DSNF than to oatmeal consumption (iAUC_0–240 h_ assessments, *p* < 0.001) and suggest a beneficial noncaloric mechanism that further encourages weight loss [5]. Subjective appetite perceptions measured along the visual analogue scale also favored DSNFs versus nonspecific formulas in a randomized blinded cross-over study of patients with T2D [6].

DSNF consumption also results in diminished glycemic variability, which correlates with better clinical outcomes, particularly among hospitalized individuals. Based on studies of diverse ICU patients, those with worsening glycemic variability had greater risk of mortality than those with better glycemic control and/or preadmission diabetes [53,54]. Among high-risk ICU patients, two different DSNFs outperformed a standard formula comparator across important clinical parameters [55]. The advanced DSNF significantly lowered insulin use (19.1 vs. 23.7 IU/day, *p* < 0.05), plasma glucose (138.6 vs. 146.1 mg/dL, *p* < 0.01), and glycemic variability (33.6 vs. 49.1 mg/dL, *p* < 0.001, through ICU days 1–28) versus the standard formula.

In adults with overweight/obesity and T2D, DSNF improved glycemic control and reduced glycemic response [56]. A1C declined −0.95% compared to −0.48% in the control group (*p* = 0.020), fasting blood glucose declined −18.47 mg/dL vs. 1.34 mg/dL among controls (*p* = 0.03), and postprandial plasma glucose was reduced −29.77 mg/dL vs.−2.64 mg/dL (*p* = 0.053) in the control group. In patients with and without diabetes admitted to ICU with critical illness and hyperglycemia, on oral or tube feedings, DSNF reduced glycemic variability (12.6% vs. 15.9%, *p* = 0.01) and insulin utilization (45.0 vs. 107 mean units over the 24-h study period, *p* = 0.02) compared to a standard control formula [57]. These findings are consistent with the most recent research that showed DSNF use among patients with T2D to replace breakfast and an afternoon snack was associated with a significant decrease in postprandial hyperglycemia, and overnight glycemic variability, as measured with continuous glucose monitoring (CGM) [58]. In a systemic review with meta-analyses of 23 studies involving 784 patients, glycemic control associated with DSNF was significantly better than that achieved with standard formulas delivered as partial meal replacement by mouth or tube feedings [4]. Results showed that DSNF compared with standard formulas consistently and significantly mitigated the rise in postprandial blood glucose by 1.03 mmol/L, lowered peak blood glucose concentrations by 1.59 mmol/L and diminished glucose AUC by 7.96 mmol in patients with type 1, type 2, or stress diabetes in multiple settings.

### 3.2. Impact of DSNF on Lipid Status

One of many RCTs of DSNF-related weight loss studied patients with obesity and T2D who were randomly assigned to a macronutrient-based dietary plan or meal replacements with DSNFs [59]. Weight loss was equivalent for patients in both meal replacement groups (−6.4% and −6.7%) but superior to those on the dietary plan (4.9%, *p* = 0.009). Fasting glucose was significantly reduced (*p* = 0.012) in the DSNF groups compared to the dietary-plan group, and lipid-lowering benefits (total cholesterol (TC) and LDL-c) were improved, TC significantly (*p* < 0.05). In another prospective study, patients with overweight/obesity and T2D were randomized to one of three arms: (A) a personal eating plan; (B) structured meal plan with DSNF; and (C) structured meal plan with DSNF and additional support [60]. Without a change in baseline activity level, those on the structured meal plans with DSNF but not the personally designed plan, experienced significant reductions in weight (*p* < 0.001), fat percentage (*p* < 0.01), waist circumference (*p* < 0.01), and A1C (*p* < 0.01) during the 16-week study term. However, only patients who received DNSF and weekly phone contact (Group C) had observed improvement in their HDL-c blood concentration (*p* < 0.05). Another masked study randomized patients with T2D to tube feeding with DSNF or an isocaloric standard feed for 12 weeks, while maintaining glycemic control [11]. Glycemic parameters (postprandial glucose response AUC [*p* = 0.008] and A1C [*p* = 0.034]) improved in the DSNF group but not in the standard-feed group. Similarly, HDL-c levels improved significantly (*p* ≤ 0.05) in the DSNF but not the standard-feed group. Other lipid parameters remained unchanged in both groups. Evidence from a systemic review and meta-analysis also showed that DSNF is effective in managing cardiometabolic parameters of disease in association with lipid changes [50]. Not only did blood glucose and A1C levels diminish in this analysis of patients with T2D, but HDL-c concentration increased. In a second systematic review and meta-analysis of pooled data, the benefits of DSNF with high-MUFA content versus a standard nutrition formula without high-MUFA were assessed for glycemic control and lipid metabolism [51]. Eighteen RCTs involving 845 patients contributed meta-analytic data. Outcomes revealed that DSNF with high-MUFA significantly decreased peak postprandial glucose, incremental glucose response, mean blood glucose, glucose variability, A1C, area under the curve (AUC) plasma insulin, mean administered insulin dose, and mean blood total triglycerides (TGs), as well as significantly increased HDLs.

### 3.3. Impact of DSNF on Hormonal and Inflammatory Markers

Mounting evidence indicates that adipocytes are not simply inert storage depots for energy but they are also constituents of active endocrine tissue that can promote either metabolic homeostasis via small adipocytes in slender people or inflammation and insulin resistance via eutopic engorged adipocytes in people with obesity, or via ectopic adipocytes in nonadipose tissue (e.g., liver, pancreas, kidney, heart, and muscle) in people with other types of abnormal adiposity [46].

Adipose tissue produces and releases adipokines and cytokines that can foster or hinder inflammation and promote or mitigate CMBCD [61]. Adiposity is therefore a state of chronic low-grade inflammation that worsens or improves by way of weight gain or loss, which can directly affect T2D and CVD. In a systematic review, weight loss was positively correlated with a decline in inflammation manifested by a decrease in C-reactive protein (CRP) concentration [62]. Pre- and postintervention (surgical, lifestyle, dietary, and/or exercise) values for mean change in CRP and weight were determined by regression analyses of pooled data from 33 different studies. For each kilogram of weight loss, CRP declined by 0.13 mg/L (Pearson correlation, r = 0.85). A near-linear correlation was noted in conjunction with lifestyle intervention.

The role of DSNF in mediating cardiometabolic risk by modulating inflammation deserves additional consideration. Considered in one small RCT (likely underpowered [*N* = 20] for quantitative changes in markers of inflammation), patients who substituted a low-glycemic DSNF replacement for a controlled isocaloric open-choice breakfast experienced a minimal but persistent reduction in CRP plasma concentration throughout the entire 12-week follow-up term of the study [63]. Open-choice dieters did not respond similarly. Clinical significance for the CRP reduction was not achieved.

The inclusion of DSNF within daily meal plans makes it difficult to discern DSNF anti-inflammatory activity independent of other dietary elements. Studying potential anti-inflammatory effects in patients fed DSNF as sole source nutrition (e.g., tube-fed) could better test this hypothesis. Such investigation of DSNF employing highly sensitive immune and enzymatic assays for CRP, F2-isoprostanes (F2-IsoPs); lipoprotein-associated phospholipase A2 (Lp-PLA2), myeloperoxidase (MPO), oxidized ApoB and LDL-c molecules (OxLDL) are still needed to better understand adipokine-cardiovascular networks and therapeutic targeting [64].

### 3.4. Impact on Blood Pressure

According to the American Heart Association, maintaining a healthy weight (18.5−24.0 kg/m^2^) lowers systolic blood pressure (SBP) by about 5 mm Hg. Clinical trials have shown that achieving and maintaining healthy blood pressure reduces the risk of CMBCD and its outcomes by 35% to 40% for stroke, 15% to 25% for myocardial infarction, and up to 64% for heart failure [65]. Few studies have focused specifically on antihypertensive outcomes brought about by DSNFs. However, one study that validated the impact of the transcultural Diabetes Nutrition Algorithm (tDNA) [44], a multifactorial approach of lifestyle interventions with DSNF for T2D, included blood pressure assessments that confirmed significant benefit [66]. At six months postintervention, A1C declined significantly in both tDNA groups: (−1.1 ± 0.1%, *p* < 0.001) and (−0.5 ± 0.1%, *p* = 0.001) but not in the control group (−0.2 ± 0.1%, *p* = NS). Likewise, weight decreased in both tDNA groups: (−6.9 ± 1.3 kg, *p* < 0.001) and (−5.3 ± 1.2 kg, *p* < 0.001) but not among controls (−0.8 ± 0.5 kg, *p* = NS). Finally, SBP was reduced by −9 ± 2 mm Hg (*p* < 0.001) and −9 ± 2 mm Hg (*p* = 0.001) in the tDNA groups but not the control group (−1 ± 2 mm Hg, *p* = NS).

In a second study of integrated interventions for patients with T2D, comparisons were drawn between a matched reference group that received traditional diabetes instruction on diet and physical activity and an intervention group that received DSNF for meal replacement along with more intense instruction and support [67]. Significant (*p* < 0.05) intergroup differences in fasting blood glucose and insulin requirement were recorded; A1C was significantly lower (*p* < 0.001) at 12- (−0.6 ± 0.1%) and 24-weeks (–0.8 ± 0.1%); mean SBP had declined by week 24 (124 ± 1 vs. 133 ± 2 mm Hg, *p* < 0.01) and diastolic blood pressure (DBP) declined (84 ± 1 vs. 89 ± 1 mm Hg, *p* < 0.01); all in favor of the intervention group. In the Look AHEAD Trial [47,68,69], an intensive lifestyle intervention (ILI) group plus DSNF was compared to a diabetes support and education (DSE) group for differences in clinical outcomes at 1-, 4-, and 8-year time horizons. Although widespread significant comparative differences were seen in the first year [68], ILI participants retained greater improvements than DSE participants at 4 years in weight (−6.15% vs. −0.88%, *p* < 0.0001), A1C (A1c, −0.36% vs. 0.09%, *p* < 0.0001), SBP (−5.33 vs. −2.97 mmHg, *p* < 0.0001), DBP (−2.92 vs. −2.48 mmHg, *p* < 0.012), and HDL-c (3.67 vs. 1.97 mg/dL, *p* < 0.0001) [47]. Clinically meaningful weight loss (≥5% in 50% of patients) was still apparent in the Look AHEAD’s ILI group in year 8 [69].

## 4. Type 1 Diabetes

Type 1 diabetes (T1D) and T2D have different etiologies and pathophysiologies that require distinct DSNF strategies. Nutritional and metabolic management of *bona fide* T1D requires exogenous insulin for patient survival. In addition, T1D and other insulinopenic states that require insulin for acceptable glycemic control (e.g., late-stage T2D, ketosis-prone diabetes, “double diabetes” (with features of both T1D and T2D), latent autoimmune diabetes of adults, postpancreatectomy or other secondary diabetes states, fibrocalculous pancreatic diabetes, and COVID-19-related diabetes) prioritize management for close synchronization between carbohydrate intake and insulinization, with chronic hyperglycemia leading to microvascular complications [70]. In contrast, T2D management, with or without severe insulinopenia, prioritizes insulin resistance targeting, which in most cases is associated with abnormal adiposity plus the need for weight loss and primarily leads to macrovascular complications within the CMBCD framework [45,46]. Inasmuch as both T1D and T2D require glycemic, lipid, blood pressure, and weight control, there is considerable overlap in their nutritional imperatives.

A 27-year (mean) follow-up assessment of the Diabetes Control and Complications Trial (DCCT) confirmed key associations between CMBCD risk factors and CVD or major clinical events (angina, revascularization, fatal or nonfatal MI, congestive heart failure, or stroke) within its T1D cohort [71]. Hyperglycemia was a critically important risk factor second only to age. For each A1C percentage point rise, there was a corresponding increase of 31% in risk for any form of CVD and a 42% rise in the risk of major events. Seven other conventional factors, such as blood pressure and dyslipidemia, were likewise related to such rising risks.

Although most DSNF studies have been conducted in T2D, a few have involved patients with T1D. In one such study, a low carbohydrate, high fat DSNF with fiber was shown to limit hyperglycemia [72]. Another study reported that the carbohydrate content in nutrition formulas significantly influenced the postprandial glycemic response and that a diabetes-specific low-carbohydrate, high-fat product attenuated responses better than nonspecific higher-carbohydrate formulas [73]. A third study noted that postprandial hyperglycemia was diminished to a greater extent with DSNF than with standard preparations, without significant variations among the three preparations in total cholesterol (TC), triglycerides, or β-hydroxybutyrate [74]. In aggregate, these and other studies previously cited herein provide preliminary evidence that DSNF may exert metabolic and clinical benefits in patients with T1D as they do in patients with T2D. Dedicated research is needed.

## 5. The Economics of DSNF Use

The benefits of DSNF extend beyond scientific and clinical considerations to key aspects of health economics that signify value. For example, among patients with malnutrition and T2D who received care in an outpatient setting, healthcare resources and costs were assessed for one year before and during DSNF utilization [52]. Health-care resources were significantly reduced in terms of fewer emergency visits (−57.7%; *p* < 0.001), hospital admissions (–54.7%; *p* < 0.001), and inpatient days (−64.1%; *p* < 0.001), year over year. Healthcare costs declined as well during the interventional period (65.6% (*p* < 0.001). Additionally, ICU utilization of DSNF in patients with T2D, compared to similarly matched non-DSNF patient controls, correlated with a significant reduction in mortality and insulin requirement, plus improved economic outcomes [75]. In this study, mortality declined (5.1% vs. 12.3%, *p* = 0.0118), as did insulin prescriptions (29.1% vs. 38.4%, *p* = 0.0269). ICU length of stay also declined (13.0 days vs. 15.1 days), but statistical significance was not achieved (*p* = 0.1843). Additionally, total ICU costs were significantly lowered for patients receiving DSNF (US$6700 vs. US$9200, *p* < 0.0001).

Quality of life (QoL) improvements have also been observed among DSNF-fed, nursing-home and community-based geriatric patients with diabetes and risk or presence of malnutrition [76]. At both 6- and 12- week assessments, in this multicentered study, DSNF was associated with improvements from baseline in BMI (*p* < 0.001), A1C (*p* < 0.001) and QoL by EQ-5D questionnaire (*p* < 0.001). A slight improvement was also observed in 12-week functional status. QoL reflects not only clinical measures but also cost utility that relates to levels of wellness when combined with economic parameters of physical and mental capabilities. As capabilities improve, so does the value of human life, generally expressed as improvements in quality-adjust life years.

## 6. Conclusions

The CMBCD model encapsulates multiple metabolic drivers, namely adiposity and dysglycemia, into one unified concept of the pathogenesis of T2D and CVD, with the explicit goal of prompting early and sustainable prevention of disease progression. With this understanding comes the realization that a prevention paradigm is paramount. The central role of adopting a vigorous lifestyle, with healthy eating patterns as a cornerstone, is evidence-based and indisputable. DSNFs can contribute to disease prevention efforts as iso- or hypocaloric meal or snack replacements, hypercaloric supplementation for malnourished patients, very-low-calorie diets, and enteral nutrition support as deemed appropriate. They have been scientifically studied and shown to confer benefits in a wide range of clinical settings. In short, DSNFs occupy a discrete place in the nutritional armamentarium for the management of diabetes and other cardiometabolic risk factors in both inpatient and outpatient settings. Recommendations for utility in clinical practice can be found in organizational guidelines and the transcultural Diabetes Nutrition Algorithm [44]. It is further hoped that ongoing research will fill any remaining knowledge gaps and help to build a better infrastructure to improve clinical action.

## Figures and Tables

**Table 1:** Actions of minerals on various aspects of cardiometabolic-based chronic disease (CMBCD).

Minerals	Impact on Dysglycemia-Based Chronic Disease (DBCD) Progression	Impact on Adiposity-Based Chronic Disease (ABCD) Progression
Calcium	Affects β-cell secretory function, insulin release, T2D complications.	
Chromium	Involved in carbohydrate metabolism and glucose homeostasis, cofactor for insulin action, component of glucose tolerance factor (GTF).	Supplements have been studied for their effects on cholesterol, heart disease risk, but results are unclear. Low levels linked to increased CVD risks.
Cobalt	Influences glycemic control, gluconeogenesis, tissue glucose uptake, GLUT-1 expression	Excessive cobalt levels cause toxicity that may lead to heart failure but overexposure is currently rare.
Copper	Affects glucose tolerance/intolerance, insulin response, and increased glucose via insulin-like activity.	Lipogenesis, hypercholesterolemia, atherosclerosis
Iodine	Correlated with thyroid stimulating hormone (TSH), which affects insulin resistance and β-cell function.	Hypothyroidism produces abnormal lipid profiles, elevated LDL-c and TC levels and raising the risk of atherosclerosis. It also weakens myocardial contractility and can cause cardiac arrhythmias.
Iron	May induce diabetes via oxidative damage to β cells, impairment of hepatic insulin extraction, and suppression of hepatic glucose production by insulin interference.	Iron deficiency may result in left ventricular dysfunction, especially when the hemoglobin level is less than 5 g/dL
Magnesium	Cofactor of many enzymes in carbohydrate metabolism. Involved in insulin metabolism, secretion, binding, and activity. Improves insulin resistance.	CVD risk, normotension state, rate and rhythm, arterial health. Low magnesium linked to CVD risk factors: hypertension, atherosclerosis with calcification
Manganese	Manganese-activated enzyme essential for the metabolism of carbohydrates, amino acids, and cholesterol. Needed for normal insulin production and secretion. Antioxidant. Inverse relationship with futureT2D	Component of potent antioxidant enzyme, manganese superoxide dismutase (MnSOD). Neutralizes the reactive oxygen species (ROS) in mitochondria. MnSOD also protects cells from inflammation.
Selenium	Antioxidant. Mimics insulin activity in models. Prevents development of diabetic complications.	In deficiency, lipid peroxides may collect in the heart, especially during ischemia, damage cell membranes, and impair calcium transport with intra-cellular accumulation
Vanadium	Affects glucose transport, glycolysis, glucose oxidation, insulin sensitivity, insulin signaling, and glycogen synthesis.	Facilitates lipid and amino acid metabolism.
Zinc	Cofactor in glucose metabolism. Required for insulin storage and cellular binding.	Cofactor for intracellular enzymes involved in lipid metabolism

Adapted from Siddiqui [30] with supplemental data and information [31,32,33,34,35,36,37]. β—beta, CVD—cardiovascular disease, GLUT—glucose transporter, LDL-c—low-density lipoprotein cholesterol, T2D—type 2 diabetes, TC—total cholesterol.

**Table 2:** Micronutrient Deficiencies Affecting T2D.

Micronutrient	Deficiency Prevalence	Deficiency Prevalence
	Obesity	T2D
B1 Thiamine	15–29%	17–79%
B6 Pyridoxine	0–11%	58–63%
B9 Folic acid	3–4%	22%
B12 Cobalamin	3–8%	22%
B7 Biotin	NA	NA
Chromium	NA	NA
Selenium	58%	NA
Vitamin A	17%	NA
Vitamin C	35–45%	13–55%
Vitamin D	80–90%	85–91%
Vitamin E	0%	0%
Zinc	14–30%	19%

Adapted from Via [38] with supplemented data [39,40,41,42]. NA—not available.

**Table 3:** Diabetes-specific nutrition formula (DSNF) studies in various diabetes and cardiometabolic clinical scenarios.

Clinical Scenario (Reference)	Cardiometabolic Risk(s)	Design	Population	Findings Intervention vs. Control	Meal Replacement(s)
[47] Outpatient. Weight loss.	CMBCD/Cardiovascular ABCD/Obesity DBCD/Diabetes, T2D	RCT	Overweight and obese patients. *N* = 5145	↓ body weight.	SlimFast (SlimFast Foods), Glucerna (Abbott Nutrition), OPTIFAST (Novartis Nutrition) and HMR (HMR, Inc., Boston, MA USA).
[48] Outpatient. Weight loss and glycemic control.	CMBCD/Cardiovascular ABCD/Obesity DBCD/Diabetes, T2D	RCT, 3 arms	Overweight and obese patients. A1C 8.7 +/− 1.5 *N* = 108	↓ A1C, body weight, body fat %, waist circumference. All *p* = 0.001	Glucerna, (Abbott Nutrition): Carb-26 g, Fat-7 g, Prot-10 g per serving Ultra Glucose Control (Metagenics) carb-27 g, fat-7 g, prot-15 g
[4] Outpatient. Glycemic control	DBCD, T2D	RCT, 2 arms	Patients with T2D. *N* = 123	Improved outcomes: SDBG (*p* = 0·005), CV (*p* = 0·002), MAGE (*p* = 0·016) and AUCpp (*p* < 0.001), SBP (*p* < 0.046)	Glucerna SR (Abbott Nutrition) carb-31 g, fat-8 g, prot-11 g per serving
[49] Inpatients and outpatients. DSNF oral and tube feeding vs. non-DSNF standard care	DBCD/Diabetes	Meta-analysis, 19 RCTs +4 non-RTC	Patients with T1D, T2D, or stress DM. *N* = 605	↓ PG, PPG, AUC-G, and insulin requirement	Various diabetes-specific formulas (containing high proportions of monounsaturated fatty acids, fructose, and fiber
[50] Varied settings. DSNF vs. standard enteral nutrition formula.	CMBCD/Cardiovascular ABCD/Obesity DBCD/Diabetes, T2D	Meta-analysis 4 RCTs +1 parallel design	Patients with T2D +/− complication. *N* = 269	↓ PPG, A1C ↑ HDL-c All *p* ≤ 0.01	Various diabetes-specific formulas with average macronutrient proportions of carb-37–55%, fat-30–45%, prot-15–19%
[51] Varied settings. High MUFA vs. standard formula.	DBCD/T2D, T1D	Meta-analysis18 RCTs	Patients with T2D, T1D, or stress DM. Enteral nutrition. *N* = 845	↓ PG, PPG, AUC-G, A1C, and insulin requirement vs. baseline. Individual results all *p* < 0.05	Various diabetes-specific formulas with MUFAs 20% of total energy or fat 40% of total energy
[52] Community or nursing home settings. Malnourished older patients. 1 year pre- and post-DSNF oral nutrition.	CMBCD/CVD ABCD DBCD/T2D	1-year retrospective, 1-year prospective observational study	Patients with T2D. *N* = 93	↓hospital admissions (−54.7%, *p* < 0.001), hospital days (−64.1%, *p* < 0.001), emergency visits (57.7%, *p* < 0.001), healthcare costs (−65.6%, *p* < 0.001) year to year.	Glucerna^®^ 1.5 Cal (Abbott Nutrition) carb-35%, fat-45%, prot-20%

(AUC-G—area under the curve glucose, AUCpp—AUC postprandial blood glucose, circ—circumference, CMBCD—cardiometabolic-based chronic disease, ABCD—adiposity-based chronic disease, CV—glucose, DBCD—dysglycemia-based chronic disease, DPP—Diabetes Prevention and Control trial, DSNF—diabetes-specific nutrition formula, GV—glycemic variability, MAGE—mean amplitude of glycemic excursions, MUFA—monounsaturated fatty acid, PG—plasma glucose, pro—prospective, RCT—randomized controlled trial, retro—retrospective, SBP—systolic blood pressure, SDBG—standard deviation, blood glucose, T1D—type 1 diabetes, T2D—type 2 diabetes, ↓—decrease, ↑—increase).

## Abbreviations

A1C—hemoglobin A1c, ABCD—adiposity-based chronic disease, Apo-B—apolipoprotein-B, AUC—area under the curve, AUC-G—area under the curve glucose, AUC-pp—area under the curve postprandial glucose, BG—blood glucose, CMBCD—cardiometabolic-based chronic disease, CRP—C-reactive protein, DSE—diabetes support and education, CVD—cardiovascular disease, DBCD—dysglycemia-based chronic disease, DBP—diastolic blood pressure, DPP—Diabetes Prevention and Control trial, DSNF—diabetes-specific nutrition formula, F2-IsoPs—F2-isoprostanes, GLP-1—glucagon-like peptide-1, GV—glycemic variability, HDL-c—high-density lipoprotein cholesterol, HHS—Department of Health and Human Services, ILI—intensive lifestyle intervention, LDL-c—low-density lipoprotein cholesterol, Lp-PLA2—lipoprotein-associated phospholipase A2, MetS—metabolic syndrome, MPO—myeloperoxidase, MUFA—monounsaturated fatty acid, OxLDL—oxidized LDL-c molecules, PG—plasma glucose, pro—prospective, PUFA—polyunsaturated fatty acid, retro—retrospective, QoL—quality of life, RCT—randomized controlled trial, SBP—systolic blood pressure, SD—standard deviation, T1D—type 1 diabetes, T2D—type 2 diabetes, tDNA—transcultural Diabetes Nutrition Algorithm, TC—total cholesterol, TG—triglyceride.

## References

[1] Institute of Medicine (2005). Dietary Reference Intakes for Energy, Carbohydrate, Fiber, Fat, Fatty Acids, Cholesterol, Protein, and Amino Acids.

[2] Arnett D.K., Blumenthal R.S., Albert M.A., Buroker A.B., Goldberger Z.D., Hahn E.J., Himmelfarb C.D., Khera A., Lloyd-Jones D., McEvoy J.W. (2019). 2019 ACC/AHA guideline on the primary prevention of cardiovascular disease: Executive summary: A report of the American College of Cardiology/American Heart Association Task Force on Clinical Practice Guidelines. Circulation.

[3] American Diabetes Association (2019). Lifestyle management: Standards of Medical Care in Diabetes 2019. Diabetes Care.

[4] Elia M., Ceriello A., Laube H., Sinclair A.J., Engfer M., Stratton R.J. (2005). Enteral nutritional support and use of diabetes-specific formulas for patients with diabetes: A systematic review and meta-analysis. Diabetes Care.

[5] Mottalib A., Abrahamson M.J., Pober D.M., Polak R., Eldib A.H., Tomah S., Ashrafzadeh S., Hamdy O. (2019). Effect of diabetes-specific nutrition formulas on satiety and hunger hormones in patients with type 2 diabetes. Nutr. Diabetes.

[6] Dávila L.A., Bermúdez V., Aparicio D., Céspedes V., Escobar M.C., Agüero S.D., Cisternas S., Costa J.D.A., Rojas D.M., Reyna N. (2019). Effect of Oral Nutritional Supplements with Sucromalt and Isomaltulose versus Standard Formula on Glycaemic Index, Entero-Insular Axis Peptides and Subjective Appetite in Patients with Type 2 Diabetes: A Randomised Cross-Over Study. Nutrients.

[7] Mechanick J.I., Farkouh M.E., Newman J.D., Garvey W.T. (2020). Cardiometabolic-Based Chronic Disease, Adiposity and Dysglycemia Drivers: JACC State-of-the-Art Review. J. Am. Coll. Cardiol..

[8] Buranapin S., Siangruangsang S., Chantapanich V., Hengjeerajarus N. (2014). The comparative study of diabetic specific formula and standard formula on postprandial plasma glucose control in type 2 DM patients. J. Med. Assoc. Thai. Chotmaihet Thangphaet.

[9] Pohl M., Mayr P., Mertl-Roetzer M., Lauster F., Lerch M., Eriksen J., Haslbeck M., Rahlfs V.W. (2005). Glycaemic control in type II diabetic tube-fed patients with a new enteral formula low in carbohydrates and high in monounsaturated fatty acids: A randomised controlled trial. Eur. J. Clin. Nutr..

[10] Lansink M., Van Laere K.M., Vendrig L., Rutten G.E. (2011). Lower postprandial glucose responses at baseline and after 4 weeks use of a diabetes-specific formula in diabetes type 2 patients. Diabetes Res. Clin. Pract..

[11] Vaisman N., Lansink M., Rouws C.H., Van Laere K.M., Segal R., Niv E., Bowling T.E., Waitzberg D.L., Morley J.E. (2009). Tube feeding with a diabetes-specific feed for 12 weeks improves glycaemic control in type 2 diabetes patients. Clin. Nutr..

[12] Evert A.B., Dennison M., Gardner C.D., Garvey W.T., Lau K.H.K., MacLeod J., Mitri J., Pereira R.F., Rawlings K., Robinson S. (2019). Nutrition Therapy for Adults With Diabetes or Prediabetes: A Consensus Report. Diabetes Care.

[13] Mansoor N., Vinknes K.J., Veierød M.B., Retterstol K. (2016). Effects of low-carbohydrate dietsv. low-fat diets on body weight and cardiovascular risk factors: A meta-analysis of randomised controlled trials. Br. J. Nutr..

[14] Sainsbury E., Kizirian N.V., Partridge S.R., Gill T., Colagiuri S., Gibson A.A. (2018). Effect of dietary carbohydrate restriction on glycemic control in adults with diabetes: A systematic review and meta-analysis. Diabetes Res. Clin. Pract..

[15] Ang M. (2016). Metabolic Response of Slowly Absorbed Carbohydrates in Type 2 Diabetes Mellitus.

[16] Veronese N., Solmi M., Caruso M.G., Giannelli G., Osella A.R., Evangelos E., Maggi S., Fontana L., Stubbs B., Tzoulaki I. (2018). Dietary fiber and health outcomes: An umbrella review of systematic reviews and meta-analyses. Am. J. Clin. Nutr..

[17] Pereira M.A., O’Reilly E., Augustsson K., Augustsson K., Fraser G.E., Goldbourt U., Heitmann B.L., Hallmans G., Knekt P., Liu S. (2004). Dietary fiber and risk of coronary heart disease: A pooled analysis of cohort studies. Arch. Intern. Med..

[18] Jakobsen M.U., O’Reilly E.J., Heitmann B.L., Pereira M.A., Bälter K., Fraser G.E., Goldbourt U., Hallmans G., Knekt P., Liu S. (2009). Major types of dietary fat and risk of coronary heart disease: A pooled analysis of 11 cohort studies. Am. J. Clin. Nutr..

[19] Mozaffarian D., Micha R., Wallace S. (2010). Effects on Coronary Heart Disease of Increasing Polyunsaturated Fat in Place of Saturated Fat: A Systematic Review and Meta-Analysis of Randomized Controlled Trials. PLoS Med..

[20] Hooper L., Summerbell C.D., Thompson R., Sills D., Roberts F.G., Moore H., Smith G.D. (2011). Reduced or modified dietary fat for preventing cardiovascular disease. Cochrane Database Syst. Rev..

[21] Kris-Etherton P.M., Pearson T.A., Wan Y., Hargrove R.L., Moriarty K., Fishell V., Etherton T.D. (1999). High-monounsaturated fatty acid diets lower both plasma cholesterol and triacylglycerol concentrations. Am. J. Clin. Nutr..

[22] Felig P., Marliss E., Cahill G.F. (1969). Plasma amino acid levels and insulin secretion in obesity. N. Engl. J. Med..

[23] Wang T.J., Larson M.G., Vasan R.S., Cheng S., Rhee E.P., McCabe E., Lewis G.D., Fox C.S., Jacques P.F., Fernandez C. (2011). Metabolite profiles and the risk of developing diabetes. Nat. Med..

[24] Ruiz-Canela M., Toledo E., Clish C.B., Hruby A., Liang L., Salas-Salvadó J., Razquin C., Corella D., Estruch R., Ros E. (2016). Plasma Branched-Chain Amino Acids and Incident Cardiovascular Disease in the PREDIMED Trial. Clin. Chem..

[25] Johnston K., Sharp P., Clifford M., Morgan L. (2005). Dietary polyphenols decrease glucose uptake by human intestinal Caco-2 cells. FEBS Lett..

[26] Jung E.H., Kim S.R., Hwang I.K., Ha T.Y. (2007). Hypoglycemic Effects of a Phenolic Acid Fraction of Rice Bran and Ferulic Acid in C57BL/KsJ-db/dbMice. J. Agric. Food Chem..

[27] Szkudelski T., Szkudelska K. (2011). Anti-diabetic effects of resveratrol. Ann. N. Y. Acad. Sci..

[28] Wallerath T., Deckert G., Ternes T., Anderson H., Li H., Witte K., Förstermann U. (2002). Resveratrol, a Polyphenolic Phytoalexin Present in Red Wine, Enhances Expression and Activity of Endothelial Nitric Oxide Synthase. Circulation.

[29] Poli A., Barbagallo C.M., Cicero A.F., Corsini A., Manzato E., Trimarco B., Bernini F., Zimetti F., Bianchi A., Canzone G. (2018). Nutraceuticals and functional foods for the control of plasma cholesterol levels. An intersociety position paper. Pharmacol. Res..

[30] Siddiqui K., Bawazeer N., Joy S.S. (2014). Variation in Macro and Trace Elements in Progression of Type 2 Diabetes. Sci. World J..

[31] Kahaly G.J. (2000). Cardiovascular and Atherogenic Aspects of Subclinical Hypothyroidism. Thyroid.

[32] Kaur B., Henry J. (2014). Micronutrient Status in Type 2 Diabetes: A Review. Adv. Food Nutr. Res..

[33] García O.P., Long K.Z., Rosado J.L. (2009). Impact of micronutrient deficiencies on obesity. Nutr. Rev..

[34] Ekpenyong C.E. (2017). Micronutrient Vitamin Deficiencies and Cardiovascular Disease Risk: Advancing Current Understanding. Eur. J. Prev. Med..

[35] Ekpenyong C.E. (2018). Micronutrient deficiency, a novel nutritional risk factor for insulin resistance and Syndrom X. Arch. Food Nutr. Sci..

[36] Cvetinovic N., Loncar G., Isakovic A.M., Von Haehling S., Doehner W., Lainscak M., Farkas J. (2019). Micronutrient Depletion in Heart Failure: Common, Clinically Relevant and Treatable. Int. J. Mol. Sci..

[37] Aasheim E.T., Hofsø D., Hjelmesæth J., Birkeland K.I., Bøhmer T. (2008). Vitamin status in morbidly obese patients: A cross-sectional study. Am. J. Clin. Nutr..

[38] Via M. (2012). The Malnutrition of Obesity: Micronutrient Deficiencies That Promote Diabetes. ISRN Endocrinol..

[39] Nix W.A., Zirwes R., Bangert V., Kaiser R.P., Schilling M., Hostalek U., Obeid R. (2015). Vitamin B status in patients with type 2 diabetes mellitus with and without incipient nephropathy. Diabetes Res. Clin. Pract..

[40] Pflipsen M.C., Oh R.C., Saguil A., Seehusen D.A., Seaquist D., Topolski R. (2009). The Prevalence of Vitamin B12 Deficiency in Patients with Type 2 Diabetes: A Cross-Sectional Study. J. Am. Board Fam. Med..

[41] Praveen D., Puvvada R.C., Vijey A.M. (2020). Association of vitamin C status in diabetes mellitus: Prevalence and predictors of vitamin C deficiency. Future J. Pharm. Sci..

[42] Daradkeh G., Zerie M., Othman M., Chandra P., Jaiosi A., Mahmood L., Alowainati B., Mohammad I., Daghash M. (2014). Zinc Status among Type (2) Diabetes Mellitus in the State of Qatar. Public Health Front..

[43] Gillespie S., Bold M.V.D. (2017). Agriculture, Food Systems, and Nutrition: Meeting the Challenge. Glob. Chall..

[44] Mechanick J.I., Marchetti A.E., Apovian C., Benchimol A.K., Bisschop P.H., Bolio-Galvis A., Hegazi R.A., Jenkins D., Mendoza E., Sanz M.L. (2012). Diabetes-Specific Nutrition Algorithm: A Transcultural Program to Optimize Diabetes and Prediabetes Care. Curr. Diabetes Rep..

[45] Mechanick J.I., Garber A.J., Grunberger G., Handelsman Y., Garvey W.T. (2018). Dysglycemia-Based Chronic Disease: An American Association of Clinical Endocrinologists Position Statement. Endocr. Pract..

[46] Mechanick J.I., Hurley D.L., Garvey W.T. (2017). Adiposity-Based Chronic Disease as a New Diagnostic Term: The American Association of Clinical Endocrinologists and American College of Endocrinology Position Statement. Endocr. Pract..

[47] Wing R.R., Look AHEAD Research Group (2010). Long-term effects of a lifestyle intervention on weight and cardiovascular risk factors in individuals with type 2 diabetes mellitus: Four-year results of the Look AHEAD trial. Arch. Intern. Med..

[48] Mottalib A., Mohd-Yusof B.N., Shehabeldin M., Pober D.M., Mitri J., Hamdy O. (2016). Impact of Diabetes-Specific Nutritional Formulas versus Oatmeal on Postprandial Glucose, Insulin, GLP-1 and Postprandial Lipidemia. Nutrients.

[49] Peng J., Lu J., Ma X., Ying L., Lu W., Zhu W., Bao Y., Zhou J. (2019). Breakfast replacement with a liquid formula improves glycaemic variability in patients with type 2 diabetes: A randomised clinical trial. Br. J. Nutr..

[50] Ojo O., Weldon S.M., Thompson T., Crockett R., Wang X.-H. (2019). The Effect of Diabetes-Specific Enteral Nutrition Formula on Cardiometabolic Parameters in Patients with Type 2 Diabetes: A Systematic Review and Meta-Analysis of Randomised Controlled Trials. Nutrients.

[51] Sanz-París A., Matía-Martín P., Martín-Palmero Á., Gómez-Candela C., Robles M.C. (2020). Diabetes-specific formulas high in monounsaturated fatty acids and metabolic outcomes in patients with diabetes or hyperglycaemia. A systematic review and meta-analysis. Clin. Nutr..

[52] Sanz-París A., Boj-Carceller D., Lardiés-Sánchez B., Perez-Fernandez L., Cruz-Jentoft A.J. (2016). Health-Care Costs, Glycemic Control and Nutritional Status in Malnourished Older Diabetics Treated with a Hypercaloric Diabetes-Specific Enteral Nutritional Formula. Nutrients.

[53] Krinsley J.S. (2009). Glycemic Variability and Mortality in Critically 111 Patients: The Impact of Diabetes. J. Diabetes Sci. Technol..

[54] Krinsley J.S. (2008). Glycemic variability: A strong independent predictor of mortality in critically ill patients. Crit. Care Med..

[55] Mesejo A., Montejo-González J.C., Vaquerizo-Alonso C., Lobo-Tamer G., Zabarte-Martinez M., Herrero-Meseguer J.I., Acosta J., Blesa-Malpica A., Martinez-Lozano F. (2015). Diabetes-specific enteral nutrition formula in hyperglycemic, mechanically ventilated, critically ill patients: A prospective, open-label, blind-randomized, multicenter study. Crit. Care.

[56] Mohan V., Kalpana N., Lakshmipriya N., Anitha P., Gayathri R., Vijayalakshmi P., Krishnaswamy K., Unnikrishnan R., Anjana R.M., Vasudevan S. (2019). A Pilot Study Evaluating the Effects of Diabetes Specific Nutrition Supplement and Lifestyle Intervention on Glycemic Control in Overweight and Obese Asian Indian Adults with Type 2 Diabetes Mellitus. J. Assoc. Phys. India.

[57] Doola R., Deane A.M., Tolcher D.M., Presneill J.J., Barrett H.L., Forbes J.M., Todd A.S., Okano S., Sturgess D.J. (2019). The effect of a low carbohydrate formula on glycaemia in critically ill enterally-fed adult patients with hyperglycaemia: A blinded randomised feasibility trial. Clin. Nutr. ESPEN.

[58] Mustad V.A., Hegazi R.A., Hustead D.S., Budiman E.S., Rueda R., Maki K., Powers M., Mechanick J.I., Bergenstal R.M., Hamdy O. (2020). Use of a diabetes-specific nutritional shake to replace a daily breakfast and afternoon snack improves glycemic responses assessed by continuous glucose monitoring in people with type 2 diabetes: A randomized clinical pilot study. BMJ Open Diabetes Res. Care.

[59] Yip I., Go V.L.W., Deshields S., Saltsman P., Bellman M., Thames G., Murray S., Wang H.-J., Elashoff R., Heber D. (2001). Liquid Meal Replacements and Glycemic Control in Obese Type 2 Diabetes Patients. Obes. Res..

[60] Mottalib A., Salsberg V., Mohd-Yusof B.N., Mohamed W., Carolan P., Pober D.M., Mitri J., Hamdy O. (2018). Effects of nutrition therapy on HbA1c and cardiovascular disease risk factors in overweight and obese patients with type 2 diabetes. Nutr. J..

[61] Lee H., Lee I.S., Choue R. (2013). Obesity, inflammation and diet. Pediatr. Gastroenterol. Hepatol. Nutr..

[62] Selvin E., Paynter N.P., Erlinger T.P. (2007). The Effect of Weight Loss on C-Reactive Protein: A Systematic Review. Arch. Intern. Med..

[63] Stenvers D.J., Schouten L.J., Jurgens J., Endert E., Kalsbeek A., Fliers E., Bisschop P.H. (2014). Breakfast replacement with a low-glycaemic response liquid formula in patients with type 2 diabetes: A randomised clinical trial. Br. J. Nutr..

[64] Mechanick J.I., Zhao S., Garvey W.T. (2016). The Adipokine-Cardiovascular-Lifestyle Network: Translation to Clinical Practice. J. Am. Coll. Cardiol..

[65] (2015). The SPRINT Research Group A Randomized Trial of Intensive versus Standard Blood-Pressure Control. N. Engl. J. Med..

[66] Chee W.S.S., Singh H.K.G., Hamdy O., I Mechanick J., Lee V.K.M., Barua A., Ali S.Z.M., Hussein Z. (2017). Structured lifestyle intervention based on a trans-cultural diabetes-specific nutrition algorithm (tDNA) in individuals with type 2 diabetes: A randomized controlled trial. BMJ Open Diabetes Res. Care.

[67] Sun J., Wang Y., Chen X., Chen Y., Feng Y., Zhang X., Pan Y., Hu T., Xu J., Du L. (2008). An integrated intervention program to control diabetes in overweight Chinese women and men with type 2 diabetes. Asia Pac. J. Clin. Nutr..

[68] (2003). The Look AHEAD Research Group Look AHEAD (Action for Health in Diabetes): Design and methods for a clinical trial of weight loss for the prevention of cardiovascular disease in type 2 diabetes. Control. Clin. Trials.

[69] The Look AHEAD Research Group (2014). Eight-year weight losses with an intensive lifestyle intervention: The look AHEAD study. Obesity.

[70] American Diabetes Association (2017). Standards of medical care in diabetes—2017. Diabetes Care.

[71] The Diabetes Control and Complications Trial/Epidemiology of Diabetes Interventions and Complications (DCCT/EDIC) Research Group (2016). Risk Factors for Cardiovascular Disease in Type 1 Diabetes. Diabetes.

[72] Peters A.L., Davidson M.B., Isaac R.M. (1989). Lack of glucose elevation after simulated tube feeding with a low-carbohydrate, high-fat enteral formula in patients with type I diabetes. Am. J. Med..

[73] Peters A.L., Davidson M.B. (1992). Effects of Various Enteral Feeding Products on Postprandial Blood Glucose Response in Patients with Type I Diabetes. J. Parenter. Enter. Nutr..

[74] Crespillo M.d.C., Olveira G., De Adana M.S.R., Rojo-Martínez G., García-Alemán J., Olvera P., Soriguer F., Muñoz A. (2003). Metabolic effects of an enteral nutrition formula for diabetes: Comparison with standard formulas in patients with type 1 diabetes. Clin. Nutr..

[75] Han Y.-Y., Lai S.-R., Partridge J.S., Wang M.Y., Sulo S., Tsao F.-W., Hegazi R. (2017). The clinical and economic impact of the use of diabetes-specific enteral formula on ICU patients with type 2 diabetes. Clin. Nutr..

[76] Matía-Martín P., Agudo F.R., Medina J.A.L., Paris A.S., Santabalbina F.T., Pascual J.R.D., Penabad L.L., Barriuso R.S., GluceNut Study Group (2019). Effectiveness of an oral diabetes-specific supplement on nutritional status, metabolic control, quality or life, and functional status in elderly patients. A multicentre study. Clin. Nutr..

